# Probing Early Tumor Response to Radiation Therapy Using Hyperpolarized [1-^13^C]pyruvate in MDA-MB-231 Xenografts

**DOI:** 10.1371/journal.pone.0056551

**Published:** 2013-02-12

**Authors:** Albert P. Chen, William Chu, Yi-Ping Gu, Charles H. Cunnhingham

**Affiliations:** 1 GE Healthcare, Toronto, Canada; 2 Department of Radiation Oncology, University of Toronto, Toronto, Canada; 3 Imaging Research, Sunnybrook Health Sciences Centre, Toronto, Canada; 4 Department of Medical Biophysics, University of Toronto, Toronto, Canada; Instituto de Investigación Sanitaria INCLIVA, Spain

## Abstract

Following radiation therapy (RT), tumor morphology may remain unchanged for days and sometimes weeks, rendering anatomical imaging methods inadequate for early detection of therapeutic response. Changes in the hyperpolarized [1-^13^C]lactate signals observed *in vivo* following injection of pre-polarized [1-^13^C]pyruvate has recently been shown to be a marker for tumor progression or early treatment response. In this study, the feasibility of using ^13^C metabolic imaging with [1-^13^C]pyruvate to detect early radiation treatment response in a breast cancer xenograft model was demonstrated *in vivo* and *in vitro*. Significant decreases in hyperpolarized [1-^13^C]lactate relative to [1-^13^C]pyruvate were observed in MDA-MB-231 tumors 96 hrs following a single dose of ionizing radiation. Histopathologic data from the treated tumors showed higher cellular apoptosis and senescence; and changes in the expression of membrane monocarboxylate transporters and lactate dehydrogenase B were also observed. Hyperpolarized ^13^C metabolic imaging may be a promising new tool to develop novel and adaptive therapeutic regimens for patients undergoing RT.

## Introduction

Accurate and non-invasive assessment of tumor response following radiation and/or chemotherapy is crucial for patient management and development of novel therapeutic regimens. Traditionally, radiation treatment planning and evaluation of tumor response are performed by anatomical imaging methods such as CT and MR imaging. Following therapy, tumor architecture may remain unchanged for days and sometimes weeks, rendering anatomical imaging methods inadequate for early detection of therapeutic response. Although PET has been utilized in recent years to detect changes in tumor glucose or amino acid metabolism, oxygenation, and proliferation following treatment [1]–[3], it is often not performed early post treatment due to confounding effects of inflammation and negative predictive values in some cancers. It is thus not clear PET would be helpful for the recent developments of hypofractionated, and adaptive RT regimens [4], [5]. There is also the risk of excessive radiation exposure with PET-CT scans if used for repeated follow up.

In the last few years, changes in the hyperpolarized [1-^13^C]lactate signals observed *in vivo* following injection of [1-^13^C]pyruvate pre-polarized via dynamic nuclear polarization (DNP) were shown to be a marker for tumor progression or early treatment response [6]–[12]. This method takes advantage of the up-regulation of glycolysis that is well known in many tumor types [13]–[15], and the recent development of the DNP-dissolution method [16], [17] that allows real time observation of cellular enzymatic reactions *in vivo* with hyperpolarized ^13^C substrates. Reduction of the flux between [1-^13^C]lactate and [1-^13^C]pyruvate observed in models of lymphoma, brain tumor and breast cancer treated with chemotherapy appeared to be linked to apoptosis [6], [8], [10]. Following radiation therapy, changes in cell proliferation capacity, growth arrest and cell death can differ greatly between different tumor models or tumors with heterogeneous phenotypes in patients [18], [19]. In this study, the feasibility of using hyperpolarized ^13^C metabolic imaging with [1-^13^C]pyruvate to detect early radiation treatment response in a breast cancer xeongraft model and the possible mechanisms of this change are investigated.

## Methods

### Cell culture and animal preparations

#### Cell preparations

The human breast cancer cell line MDA-MB-231 (kindly provided by Dr. G. Czarnota, Sunnybrook Health Sciences Centre; originally obtained from ATCC, Bethesda, MD, USA) was grown in high glucose RPMI-1640 containing 10% FBS, 100 IU penicillin and 100 µg streptomycin/ml (Wisent, St-Bruno, Quebec, Canada), and mouse endothelial MS1 cells (kindly provided by Dr. D. Dumont, Sunnybrook Health Sciences Centre; originally obtained from ATCC, Bethesda, MD, USA) were grown in Dulbecco's modified Eagle's medium containing 10% FBS (Wisent) in a 37°C humidified incubator containing 5% CO_2_ in air. MDA-MB-231 cells were sub-cultured 1∶8 by trypsinization upon reaching 95% confluence, and MS1 cells were sub-cultured 1∶5 by trypsinization right after reaching 100% confluence. For implantation, 90% confluent cells were harvested by trypsinization, washed in PBS (phosphoate-buffered saline) and assessed for viability by trypan blue dye exclusion. The cells were re-suspended in Matrigel (BD Biosciences, Finger Lakes, NJ) before xenograft implantation.

#### Tumor preparations and treatment

Animal experiments in this study were approved by the animal care and use committee at Sunnybrook Health Sciences Centre. Male RNU nude rat, 6–7 weeks old (Harlan laboratories, Mississauga, ON, Canada) were housed 2 per cage in sterilized rat cages, maintained at constant temperature and humidity and fed with regular autoclaved chow diet with water ad libitum. Four days prior to the tumor cell implantation, the rats were fasted overnight and received a single dose of 500 cGy total body irradiation [20] which was administered using a ^137^Cs irradiator (Mark 68A, JL Shepherd, San Fernando, CA) delivering approximately 74 cGy/min. The whole-body, low dose irradiation helps to improve tumor take rates for human cancer models in nude rats, as previously reported [20], [21]. Rats were placed in a sterile mouse cage during irradiation.

For implantation, rats were anesthetized using isoflurane. The skin over the injection site was cleaned with 70% ethanol. A 200 µl of cell suspension containing 4×10^6^ MDA-MB-231 and 0.4×10^6^ MS1 cells [22], [23] were injected into the subcutaneous tissue of the rat hind leg using a tuberculin syringe with a 28 gauge needle. The purpose of adding the MS1 endothelial cells was to improve tumor perfusion by creating a network of tubules within the tumor as in [19]. At two-week intervals after injection, the rats were monitored for tumor growth. The tumors became visible lumps at 4 to 6 weeks after injection.

When each tumor reached approximately 1.5 cm in the largest dimension, the rat was either scanned as a control or treated with radiation. The average duration from tumor cell implantation to imaging was 48 days (stdev. = 11) for the control group and 51 days (stdev. = 9) for the treatment group. For the radiation treatment, the rats were anesthetized using a mixture of Ketamine and Xylazine at 7.5 mg and 1 mg per 100 g body weight respectively. The tumors were exposed to ionizing radiation using a model CP160 160-kVp x-ray system (Faxitron X-ray Corp., Wheeling, IL, USA) [24]. Radiation treatments were given at a dosage of 8 Gy to one side and another 8 Gy at the opposite side of the tumor (lead shielding was used to protect the animal from radiation exposure beyond the tumor). Tumors treated with radiation were scanned 96 hours after treatment. A total of 20 animals were imaged (10 treated and 10 untreated) in this study.

### Hyperpolarized ^13^C MR imaging and spectroscopy

#### Hardware and agent

All studies were performed using a 3T GE MR750 scanner (GE Healthcare, Waukesha, WI) and a micro-strip dual-tuned ^1^H-^13^C volume coil (8 cm I.D, Magvale LLC, San Francisco, CA). A HyperSense DNP polarizer (Oxford Instruments, Abingdon, UK) was used to polarize the substrate. Neat [1-^13^C]pyruvic acid (Isotec, Miamisburg, OH) doped with 15 mM of OX63 radical and 1 mM Gd chelate (Prohance®, Bracco International) was polarized and dissolved using the established protocol [25]. The polarization achieved in solution was not measured in these studies, but were approximately 15–20% based on previous studies using similar equipment and protocol [26], [27].

#### ^13^C MRI experiments in vivo

Time-resolved ^13^C metabolic imaging was performed on tumor bearing rats *in vivo* using an EPI sequence incorporating spectral-spatial excitation [28] with (5 mm)^3^ isotropic spatial resolution and 5 s temporal resolution from a 3D volume that included the kidneys and tumor (FOV: 8 cm×8 cm×6 cm). Injection of 2 ml/80 mM pre-polarized [1-^13^C]pyruvate was performed (0.2 ml per second) into the tail vein for each study. The data acquisition was started at the beginning of the substrate injection. Resonances of [1-^13^C]lactate, [1-^13^C]pyruvate and ^13^C-urea (reference phantom) were excited and sampled sequentially during each 5 s interval [29]. The nominal effective tip angle (after the 12 excitations required to image each volume) at each time point was 60 and 9 degrees for lactate and pyruvate, respectively (RF pulse amplitude were modulated to achieve the different tip angle). A smaller tip angle was used for [1-^13^C]pyruvate to prevent premature saturation of the pre-polarized signal [29], [30]. The duration of data acquisition was 60 s (12 temporally resolved images for each metabolite). This imaging approach allows the imaging window to be extended beyond a shorter (10–20 s), fixed window centered around a presumed temporal maximum of metabolites used in prior ^13^C MR spectroscopic imaging studies [6]–[8]. The magnitude images of ^13^C pyruvate and lactate were reconstructed by the default MR scanner software (k-space data were zero filled to 128×128 in plane matrix prior to Fourier transform). ROIs of tumors (from two consecutive 5 mm axial slices in the SI center of the tumor) and left kidney (one 5 mm axial slice) were drawn on the ^1^H anatomical images using OsiriX DICOM image viewer (http://www.osirix-viewer.com/). The ^13^C pyruvate and lactate images were overlay on the anatomical images and lactate and pyruvate signals from the tumor and kidney ROIs were measured and corrected for the different nominal tip angles used (pyruvate signal amplitudes were multiplied by sin(60)/sin(9)) but not by the phantom signal. The summed data from all time points (i.e. area under the curve) were used for analysis. T_2_-weighted ^1^H anatomical images were acquired using a fast spin-echo (FSE) pulse sequence (Axial: FOV = 12 cm, 256×192 matrix, 5 mm slice thickness, from the same slice locations as the ^13^C images; Coronal: FOV = 12 cm, 256×192 matrix, 3 mm slice thickness) for localization and volume measurements of the tumors. To estimate the tumor volume, ROIs around the tumor were drawn on consecutive slices of axial T_2_-weighted ^1^H anatomical images using OsiriX DICOM image viewer, tumor volume was calculated as summed ROI area multiplied by the slice thickness.

#### ^13^C MRS experiments in vitro

Viable cell suspensions were prepared using a previously described protocol [31]. Time resolved ^13^C MR spectroscopy experiments were performed on untreated (n = 2) and radiation treated (96 hrs post treatment, n = 2) MDA-MB-231 cells following infusion of 600 µl of pre-polarized 40 mM [1-^13^C]pyruvate/20 mM sodium lactate (not ^13^C enriched) solution into the cell suspension [8]. The addition of non-enriched lactate to the hyperpolarized solution was necessary to increase the detection limit for [1-^13^C]lactate in these experiments, as it provided a larger lactate pool in the cell suspension to be exchanged with the substrate (as demonstrated in Ref. 7). The protocol still allows investigation of changes in apparent pyruvate – lactate flux due to alternation in LDH expression or availability of co-factor NAD(H). A pulse-acquire pulse sequence was used with 10° tip angle and 3 s TR (5000 Hz/2048 pts readout).

### Ex vivo and in vitro assays

#### Immunohistochemistry of the tumors

The tumors were harvested and fixed in 10% neutralized formalin immediately after MRI scanning. Terminal deoxynucleotidyl transferase mediated dUTP-biotin nick end labeling (TUNEL) was used to assess apoptosis in the tumors [32]. TUNEL data were expressed as percentages of positively stained cells from six 40× fields per tumor slide.

Senescence-associated β-galactosidase (SA-β-Gal) was used as a biomarker for cellular senescence [33]. For β-galactosidase staining, frozen tissues were sectioned at 8 mm thick and fixed and stained with staining solution mix containing X-gal at PH 6.0, and then the slides were rinsed with distilled water, dehydrated through alcohol, cleared in Xylene and mounted with paramount. SA-β-galactosidase data were calculated as the average percentage of positively stained cells from six fields that each contained at least 100 cells.

To assess tumor vascularity, cluster of differentiation 31 (CD31) staining was performed [34]–[36]. For each tumor, one 5 µm tissue section was cut and deparaffinised in xylene, rehydrated in a graded series of ethanol solutions, and heated in a microwave oven in 0.01 M sodium citrate buffer (pH 6.0) for 10 minutes for antigen retrieval. Specimens were blocked in 10 percent normal goat serum (Sigma-Aldrich) for 20 min. The sections were then incubated with a 1∶50 diluted mouse CD31 monoclonal antibody (Santa Cruz Biotechnology, Santa Cruz CA), at room temperature for 1 h, and then incubated with FITC labelled goat anti-rabbit antibody (Santa Cruz Biotechnology). Negative controls were produced by eliminating the primary antibodies from the diluents. After washing in PBS with 0.05% Tween20, the slides were counter-stained with DAPI (Sigma-Aldrich). Six fields at 200× magnification per section, randomly selected from non-necrotic regions of each tumors were examined with a fluorescent microscope (Zeiss Axiovert 200 m, Carl Zeiss Microscopy, Peabody MA). All blood vessels positive for CD31 and with distinct (slot-like, tubular, or polymorphous) lumens were counted. Micro-vessel density (MVD) was expressed as number of positive lumens for per field.

#### Cell apoptosis and senescence assays following radiation in vitro

MDA-MB-231 cells were harvested by standard trypsinization, washed with PBS and re-suspended in complete medium. The cells were seeded at 0.3×10^6^ cell/5 ml medium/plate (60 mm), grown overnight and then irradiated with 16 Gy (same system as used to treat the tumors). The cells were placed back into the incubator immediately after irradiation. For apoptosis detection, cells (96 hrs post radiation treatment, n = 5; and untreated cells, n = 4) were gently trypsinized and washed once in PBS and 0.1×10^6^ cells were stained with Annexin 5 and PI using the FITC Annexin5 apoptosis detection kit (BD Biosciences) according to manufacturer's direction, followed by flow cytometry [37].

SA-β-Gal expression was measured using a standard senescence detection kit (BD Biosciences) according to the manufacturer's instructions. In brief, culture media were removed and the cells were then washed once with PBS and fixed with the fixation solution for 15 min at room temperature. After two additional washes with PBS, the staining solution containing 1 mg/ml 5-bromo-4-chloro-3-indolyl-β-d-galactoside was added to each well. Cells (n = 4 for both control and treated cells) were incubated at 37°C overnight and then observed under a microscope for development of blue color. The percentage of blue stained cells versus total cells was measured by choosing 6 random microscopic fields that had at least 100 cells for each dataset.

To estimate change in cell size post treatment, trypsinized cells were loaded into a hemocytometer and images at 200× amplification were acquired using a Leica DM-LB2 microscope equipped with DFC480 camera (Leica Microsystems, Wetzlar, Germany). Cell lengths of 100 control and 100 treated cells were measured using ImageJ software (National Institute of Health), calibrated based on the known size of the hemocytometer grid. Changes in protein contents of the cells after treatment were determined with a Bio-Rad protein assay kit (Bio-Rad, Hercules, CA, USA).

#### Western Blot Analysis

Anti-human monocarboxylate transporter 4 (MCT4) antibodies were purchased from Santa Cruz Biotech.Inc (Santa Cruz, CA, USA). Lactate dehydrogenase A (LDHA) and Glyceraldehyde 3-phosphate dehydrogenase (GADPH) were purchased from Abcam Inc (Cambridge, MA). Lactate dehydrogenase B (LDHB) was obtained from Proteintech (Chicago, IL, USA). Horseradish peroxidase (HRP)-conjugated secondary antibodies were obtained from GE Healthcare (GE Healthcare Bio-Sciences Corp, USA). HIF1-alpha was obtained from Santa Cruz Biotech.Inc (Santa Cruz, CA, USA).

Tumor tissues were homogenized with a PowerGen Model 125 homogenizer (Fisher Scientific) with ice-cold RIPA buffer (50 mM Tris-HCl, pH 8.0, 150 mM NaCl, 1% Nonidet P40, 0.5% sodium deoxycholate, 0.1% sodium dodecylsulfate, 2 mM ethylenediaminetetraacetic acid, 1 mM phenylmethylsulfonylfluoride, 1 mM NaVO_3_, 1 mM NaF) and kept on ice for 20 minutes. The lysates were clarified by centrifugation at 16,000 g for 10 minutes at 4°C. The protein concentration was determined with a Bio-Rad protein assay kit (Bio-Rad, Hercules, CA, USA).

Cells were harvested and washed twice with PBS and lysed with ice-cold RIPA buffer. To each well, 10 µg of protein from each sample of cell lysate or 15 µg of protein from each sample of tumor tissue lysate was loaded to 12% SDS-PAGE gel with loading buffer. Separated proteins were transferred electrophoretically from gels to Immobilon-P membranes (Millipore, Bedford, MA). Membranes were incubated for 1 hour at room temperature in blocking buffer (20 mmol/L Tris - pH7.4-, 137 mmol/L NaCl, 5% dry skim milk) followed by 2 hours of incubation with primary antibodies: LDHA (1∶2,000), LDHB (1∶2,000), MCT4 (1∶300), HIF1-α (1∶100) and 1 hour with HRP-conjugated secondary antibodies with 1∶4,000 dilution. Reactive bands were visualized with ECL Western Blotting Detection Reagents (GE Healthcare Bio-Sciences Corp.).

## Results

**Figure 1:**
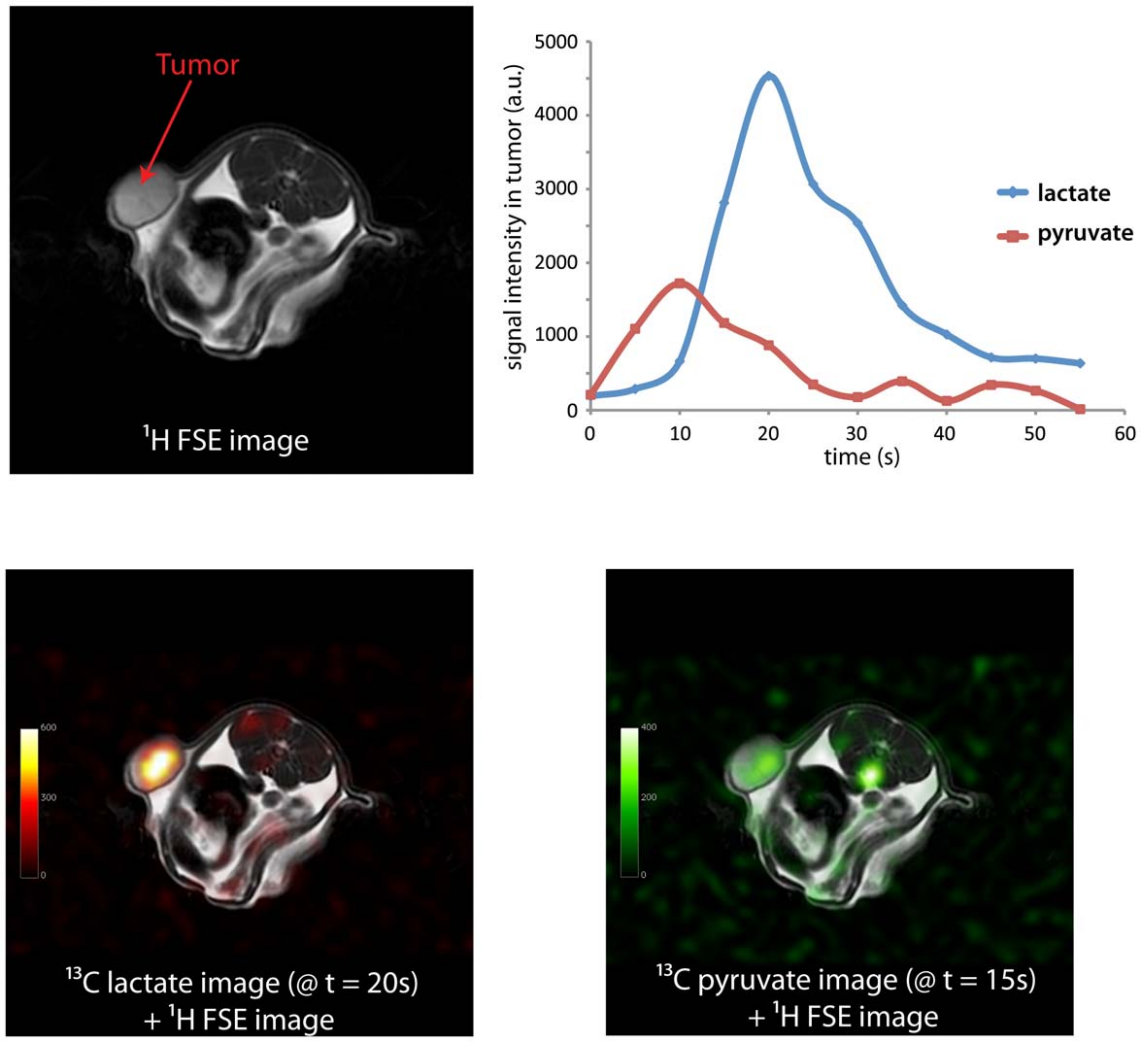
Representative T2-weighted ^1^H anatomical image (upper left), hyperpolarized [1-^13^C]lactate image and [1-^13^C]pyruvate image (lower left and right, respectively) acquired from the MDA-MB-231 rat xenograft model. Signals from lactate and pyruvate measured from the tumor were also plotted as a function of time (upper right).

Hyperpolarized [1-^13^C]lactate and [1-^13^C]pyruvate signals were observed in the tumors following injections of pre-polarized [1-^13^C]pyruvate in the rat MDA-MB-231 xenograft model (Fig. 1). Much higher [1-^13^C]lactate signal as compared to [1-^13^C]pyruvate signal was observed in the tumors, primarily due to the larger tip-angle used to acquire the lactate images. In axial image slices through the tumors, most of the lactate signal observed was within the tumors while a substantial substrate signal can be observed in the area of major vascular structures. Similar to prior dynamic ^13^C MRS data acquired from mice tumors [7], [8], different time courses were observed for pyruvate and lactate (Fig. 1, upper right). The substrate signal reached a maximum around the time the injection ended (average 11.5 s for control tumors and 9.75 s for the treated tumors, which was not significantly different, P>0.2, Student's t-test) and the [1-^13^C]lactate signal continued to increase after the end of the bolus and then decayed due to T1 relaxation and, possibly, efflux.

**Figure 2:**
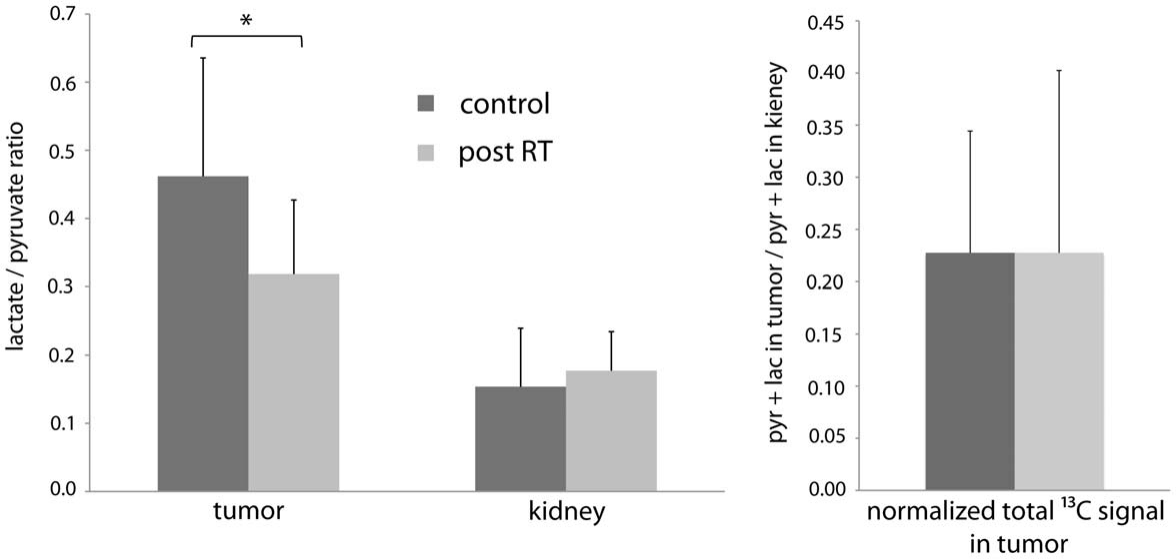
Parameters obtained from hyperpolarized ^13^C metabolic imaging of MDA-MB-231 tumors. Left: ratio of lactate to pyruvate signals measured in the rat tumors and kidneys (* P<0.05). Right, Total ^13^C signals in the tumors (pyruvate+lactate) normalized by total ^13^C signals in the kidneys.

Significantly lower average lactate to pyruvate ratios were observed in radiation treated tumors as compared to the control tumors (0.32 vs. 0.46, p<0.05, Fig. 2, left), while slightly higher lactate over pyruvate ratios were observed in the rat kidneys for the treated animals (0.18 vs. 0.15, not statistically significant, Fig. 2). Total ^13^C signal in the tumor (pyruvate+lactate) normalized by the total ^13^C signal in the kidney were similar between the control and the treated animals (Fig. 2 right). Average tumor volumes for the treated cohort were larger than for the control cohort at the time of imaging (6.6 ml vs. 4.7 ml) but the difference was not significant (P>0.3).

**Figure 3:**
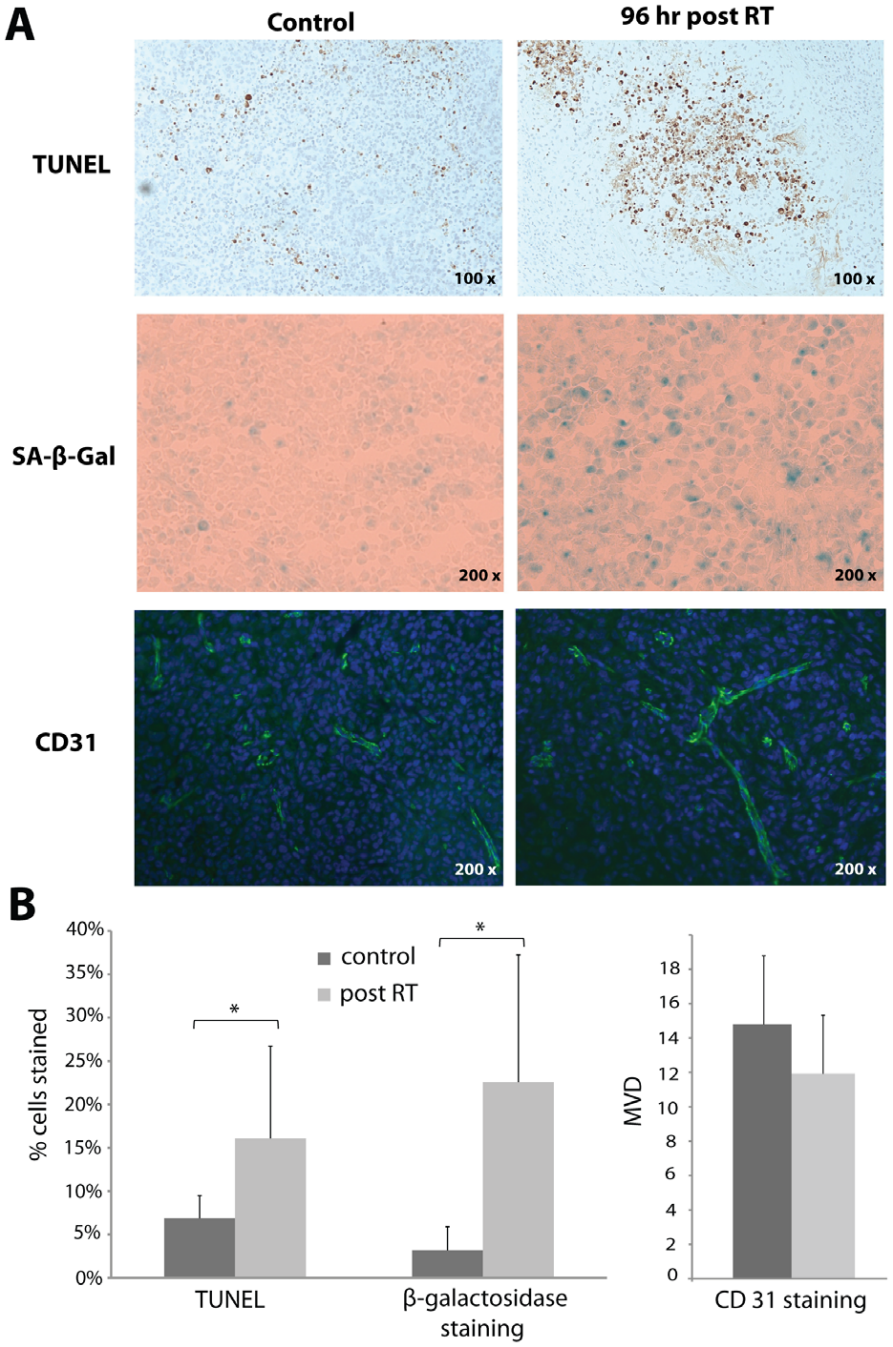
Apoptosis, senescence and vascularity of the tumors were assessed by TUNEL, SA-β-galactosidase and CD31 staining, respectively. A) Representative photographs of TUNEL, β-galactosidase and CD31 staining of control or radiation treated MDA-MB-231 tumors. B) Significantly increased cell apoptosis and senescence were observed for tumors treated with radiation (* p<0.05). Lower MVD was observed for the treated tumors but the difference was not significant.

TUNEL and β-galactosidase assays were performed on harvested tumors following the imaging studies to assess apoptosis and senescence, respectively. A significant increase in apoptosis was observed in the irradiated tumors as compared to the untreated tumors (16.1% vs. 6.5%, p<0.05, Fig. 3). A larger and also significant increase in β-galactosidase staining was found in the radiation treated tumors as compared to the controls (22.6% vs. 3.2%, p<0.05, Fig. 3). CD31 staining was also performed to assess any changes in tumor microvasculature post radiation therapy. Slightly lower MVD was observed in radiation treated tumors as compared to controls, and the difference was not statistically significantly (14.7 vs. 12.0, Fig. 3). Long segments of the tubules formed by the MS1 cells [23] were observed in the tumor histopathologic slides but showed virtually no TUNEL or β-galactosidase staining, both in the radiation treated tumors and the controls, indicating that the observed changes were not likely influenced by radiation response of the MS1 cells.

**Figure 4:**
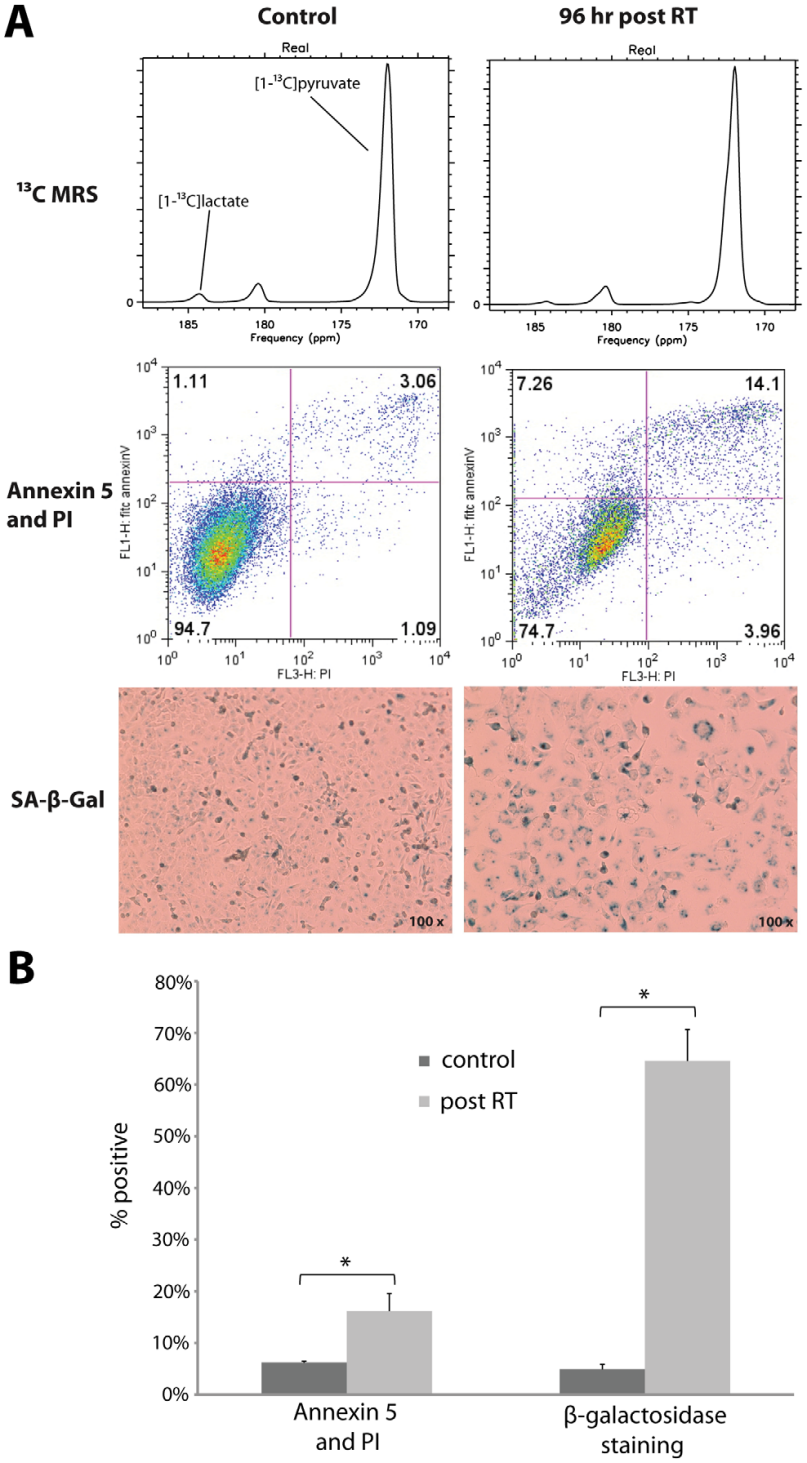
Cell apoptosis, senescence and flux between pyruvate and lactate were investigated in MDA-MB-231 cells after radiation treatment *in vitro*. A) Representative flow cytometry data from the Annexin 5 and PI assay, β-galactosidase staining and ^13^C MRS spectra from cells at 96 hours post 16 Gy radiation as well as control cells are shown. B) Significant increase in cell apoptosis and senescence were observed in treated cells as compared to control cells (* P<0.05).

The contribution of ionizing radiation to cell apoptosis and senescence of MDA-MB-231 cells at 96 hrs post treatment was also studied *in vitro*. The apoptosis assay on treated and control cells demonstrated an increase in apoptosis after radiation (16.2% vs. 4.2%, Fig. 4). Similar to the tumors, a large increase in β-galactosidase positive cells were observed in treated cells as compared to control cells (64.6% vs. 4.9%, Fig. 4). The radiation treated MDA-MB-231 cells also appeared morphologically to be much larger than the controls cells, likely the result of cell senescence [38]. The average length of the cells increased significantly from 11.1 µm (stdev. = 2.7, n = 100) to 24.9 µm (stdev. = 8.2, n = 100) with radiation treatment (p<0.00001). The protein content increased five fold from 0.23 mg (stdev. = 0.035, n = 3) to 1.16 mg (stdev. = 0.125, n = 4) per 1×10^6^ cells post radiation (p<0.05). Changes in metabolic flux between pyruvate and lactate in the cell cultures were also investigated by ^13^C MRS after the cell suspensions were perfused with pre-polarized [1-^13^C]pyruvate. Lower lactate signal relative to the substrate signal was observed in the treated cells (3×10^7^ cells, total lactate/pyruvate ratio = 0.11 and 0.14) as compared to controls (1.5×10^8^ cells, total lactate/pyruvate ratio = 0.27 and 0.39). The smaller number of post-treatment cells used in these experiments was chosen to keep the protein content constant.

**Figure 5:**
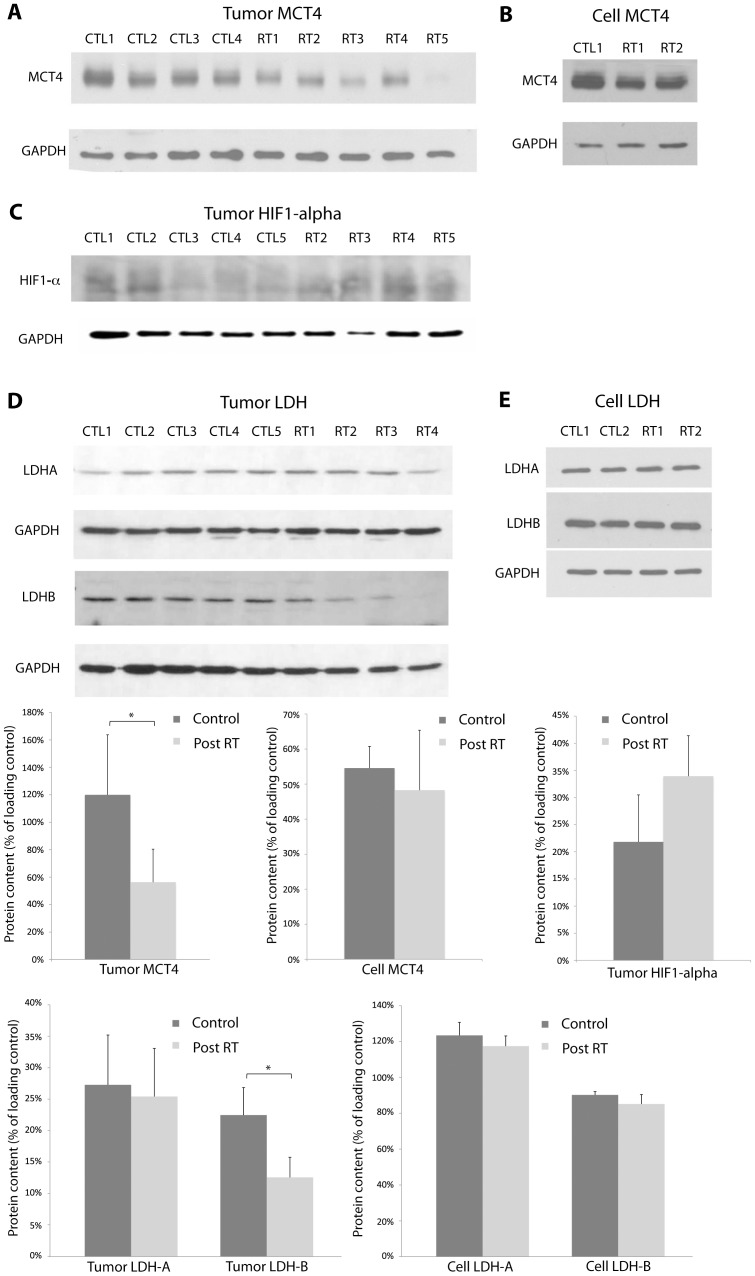
Western blot analysis was used to assess cell membrane monocarboxylate transport and lactate dehydrogenase levels. Tissue hypoxia in the tumors was also assessed by HIF1-α expression. A) Western blots showed a decrease in MCT4 expression in MDA-MB-231 tumors radiation treated with radiation as compared to controls. The difference was significant (* P<0.03). B) A small decrease in MCT4 expression was observed in treated MDA-MB-231 cells *in vitro*. C) HIF1-α expression was higher for the treated tumors, but the difference was not significant. D) Expressions of lactate dehydrogenase isoforms B were significantly lower in treated tumors. E) Little changes were found for expressions of LDH-A and LDH-B in treated and control cells. GAPDH was used as a loading control.

Western blot analysis was used to assess cell membrane monocarboxylate transport and lactate dehydrogenase levels to determine the association of these proteins with the observed decrease in metabolic flux between pyruvate and lactate. Tissue hypoxia in the tumors was also assessed by HIF1-α expression. In both radiation treated MDA-MB-231 tumors *in vivo* and cell *in vitro*, decreases in MCT4 expression were observed (Fig. 5. A and B) and the decrease in tumors was significant (P<0.03). An increase was found in HIF1-α expression for the treated tumors (Fig. 5. C), but the difference was not significant. Expressions of LDHA appeared unchanged between treated tumors and controls but significantly decreased LDHB expression was observed for the treated tumors (Fig. 5. D). Very little difference was found for both LDHA and LDHB expressions between the treated and control cells *in vitro*.

## Discussion

By detecting changes in metabolic flux between key intermediates of cellular metabolism, hyperpolarized ^13^C metabolic imaging is a promising new tool for assessment of tumor grade and early response to therapies [6]–[11]. The detection of early response non-invasively may facilitate adaptive radiation therapy either alone or in conjunction with chemotherapy. With the emergence of hypofractionated and ablative radiotherapy regimens, and the advent of MR-guided linear accelerators, this technique offers the potential for functional tumor localization and delineation, and real-time tumour response assessment.

In this study, we demonstrated that significant a decrease in hyperpolarized [1-^13^C]lactate (relative to the [1-^13^C]pyruvate substrate signals) *in vivo* in a MDA-MB-231 tumor model can be observed 96 hours after a single dose of 16 Gy ionizing radiation. Assuming consistent dose and delivery of the tracer into the tumor cells, this change in relative lactate and pyruvate signal in the tissue can be used as a marker of change in the metabolic flux between pyruvate and lactate [8]. The fast 3D volumetric imaging method enabled acquisitions of temporal and spatial imaging of the injected pre-polarized substrate and its metabolites throughout the rat torso and abdomen. Since the relative lactate to pyruvate signals measured in the kidneys showed little difference between the treated and the control animals, it is likely that the decrease in apparent flux between pyruvate and lactate was the result of the radiation therapy to the tumors. Although DCE-MR imaging was not performed in this study, the temporal dynamics of the substrate bolus signal in the tumor were recorded at similar time points and temporal resolution compared to DCE-MR imaging (the time from injection to the peak of the pyruvate signal in the tumors), and was found to be similar between the two groups. The total cumulative ^13^C signals in the tumor relative to the kidney (pyruvate+lactate from all time points) were also very similar between the two groups. Since the hyperpolarized ^13^C substrate was administered intravenously, a decrease in tumor perfusion would presumably result in lower overall ^13^C signal in the tumor. Thus the observed changes in apparent metabolic flux were likely not caused by any substantial changes in tumor perfusion, but more studies are needed to confirm this.

Similar to prior studies investigating the decrease in the [1-^13^C]lactate in tumors following chemotherapy, the metabolic changes observed in MDA-MB-231 tumors in this study were associated with an increase in apoptosis at 96 hours after the single dose of ionizing radiation. In addition to the increase in cell apoptosis, the radiation therapy also induced a significant degree of senescence in these tumors [38], [39]. The cell senescence may have contributed to the apparent increase in tumor size in the treated cohort, as senescent cells are generally much larger in size as compared to proliferating cells [38]. However, it is also possible that the slightly longer period from tumor cell implantation to imaging for the treatment group has also contributed to this difference in tumor size. The potential impact of the therapy-induced apoptosis and senescence on the flux between pyruvate and lactate measured was also investigated *in vitro* in the same cell line. Similar to the tumors, at 96 hours after a dose16 Gy radiation, MDA-MB-231 cells *in vitro* showed a small increase in apoptosis but also the majority became senescent. Since we observed a greater than 40% reduction in the apparent flux between pyruvate and lactate in tumors, while only 10% more of tumor cells become apoptotic (more than 80% of the tumor cells were still non-apoptotic post treatment), thus, the radiation-induced cell senescence and additional cellular and tissue changes may have also contributed to the observed decrease in metabolic flux.

p53 activation and downstream regulation of metabolism is observed in some tumors with ionization radiation induced accelerated senescence [40], [41]. The MDA-MB-231 cell line used in our study has mutated p53 gene and thus p53 activation was not likely the mechanism for the decreased metabolic flux observed. But it is possible that GAPDH intracellular translocation to the nucleus associated with DNA damage may contribute to a decrease in cytosolic GAPDH activity [42], [43], and lead to a reduction of NAD+/NADH pool, and thus a reduction of pyruvate/lactate flux. It is also known that transformed neoplastic cells that lack functional p53 still have the capacity for accelerated senescence through other tumor suppressor or cell-cycle regulation pathways [39], [44]. Although we observed a similar amount of apoptosis for the MDA-MB-231 cells in culture at 4 days post a 16 Gy dose of radiation as a prior study at 5 days post a 10 Gy dose [41], much higher cell senescence was observed in our study. The larger dose of radiation used in this study may be a possible reason behind this discrepancy. Further investigations are needed to elucidate the possible link between the radiation-induced senescence and metabolic changes observed in this study.

Other factors that may have directly impacted the observed change in apparent metabolic flux between hyperpolarized pyruvate and lactate such as tumor vascularity, tumor hypoxia, cellular membrane transport of the injected substrate, and the enzymes that facilitate this metabolic reaction were also investigated in this study. The small decrease in MVD and the significantly lower MCT4 expression in treated tumors suggested that less of the injected hyperpolarized pyruvate reached and entered the tumors cells to be metabolized, thus contributing to the lower metabolite (lactate) to substrate ratios in the treated group. LDH-B expression was also found to have significantly decreased post radiation and likely influenced the apparent metabolic flux after treatment. Although increased HIF1-α expression was observed post treatment and hypoxia can be associated with higher cellular lactate concentration (and potentially higher lactate to pyruvate ratios), the impact of any increase in tissue hypoxia on the observed imaging contrast was likely small as compared to other tissue and molecular changes, since *lower* lactate to pyruvate ratio was observed post therapy.

Tumor response to ionizing radiation is a complex and dynamic phenomenon, and is a subject of active research. While efforts were made in this study to correlate the apparent change in metabolic flux between pyruvate and lactate to the cellular and molecular markers that have more immediate link to the observed imaging contrast (the transport and metabolism of the injected pyurvate), other tissue, cellular, and molecular changes associated with radiation response at different stages post treatment may also be investigated in the future to provide better understanding of the imaging findings and provide other potential targets for hyperpolarized ^13^C metabolic imaging. Studies that compare the current method to other imaging techniques such as DCE-MR, various PET probes and other hyperpolarized ^13^C substrates at early time points post treatment would also be valuable to help develop protocols to characterize early therapy response of breast tumor.

## Conclusion

Detection of an early (96 hour) response to a single dose of radiation therapy *in vivo* in a MDA-MB-231 tumor model was demonstrated using hyperpolarized [1-^13^C]pyruvate in this study. It was also shown that the decrease in flux between pyruvate and lactate was likely associated with radiation-induced apoptosis and senescence, as well as changes in cellular membrane transport of monocarboxylic acid and lactate dehydrogenase expression. Future studies are needed to determine the relative contribution of the therapy-induced apoptosis, senescence, changes in monocarboxylate transporters, and LDH expressions to the observed metabolic changes.

## References

[1] Bussink J, Kaanders JH, van der Graaf WT, Oyen WJ (2011) PET-CT for radiotherapy treatment planning and response monitoring in solid tumors. Nat Rev Clin Oncol 8: 233–242. 21263464 10.1038/nrclinonc.2010.218

[2] Messa C, Di Muzio N, Picchio M, Gilardi MC, Bettinardi V, et al (2006) PET/CT and radiotherapy. Q J Nucl Med Mol Imaging 50: 4–14. 16557199

[3] Schoder H, Fury M, Lee N, Kraus D (2009) PET monitoring of therapy response in head and neck squamous cell carcinoma. J Nucl Med 50 Suppl 1: 74S–88S. 19380408 10.2967/jnumed.108.057208

[4] Hurkmans CW, Dijckmans I, Reijnen M, van der Leer J, van Vliet-Vroegindeweij C, et al (2012) Adaptive radiation therapy for breast IMRT-simultaneously integrated boost: Three-year clinical experience. Radiother Oncol 103: 183–187. 22280808 10.1016/j.radonc.2011.12.014

[5] Ko EC, Forsythe K, Buckstein M, Kao J, Rosenstein BS (2011) Radiobiological rationale and clinical implications of hypofractionated radiation therapy. Cancer/Radiotherapie 15: 221–229. 10.1016/j.canrad.2010.12.007 21514198

[6] Park I, Bok R, Ozawa T, Phillips JJ, James CD, et al (2011) Detection of early response to temozolomide treatment in brain tumors using hyperpolarized 13C MR metabolic imaging. J Magn Reson Imaging 33: 1284–1290. 21590996 10.1002/jmri.22563 PMC4983457

[7] Albers M, Bok R, Chen AP, Cunningham CH, Zierhut ML, et al (2007) Hyperpolarized 13C Lactate, Pyruvate, and Alanine: Noninvasive Biomarkers for Prostate Cancer Detection and Grading. Cancer Res 68: 8607–8615. 10.1158/0008-5472.CAN-08-0749 PMC2829248 18922937

[8] Day SE, Kettunen MI, Gallagher FA, Hu D, Lerche M, et al (2007) Detecting tumor response to treatment using hyperpolarized 13C magnetic resonance imaging and spectroscopy. Nature Medicine 13: 1382–1387. 10.1038/nm1650 17965722

[9] Bohndiek SE, Kettunen MI, Hu D-e, Brindle KM (2012) Hyperpolarized 13C Spectroscopy Detects Early Changes in Tumor Vasculature and Metabolism after VEGF Neutralization. Cancer Research 72: 854–864. 22223844 10.1158/0008-5472.CAN-11-2795 PMC3378497

[10] Witney TH, Kettunen MI, Hu D-e, Gallagher FA, Bohndiek SE, et al (2010) Detecting treatment response in a model of human breast adenocarcinoma using hyperpolarised [1-13C]pyruvate and [1,4-13C2]fumarate. British Journal of Cancer 103: 1400–1406. 20924379 10.1038/sj.bjc.6605945 PMC2990617

[11] Day SE, Kettunen MI, Cherukuri MK, Mitchell JB, Lizak MJ, et al (2011) Detecting response of rat C6 glioma tumors to radiotherapy using hyperpolarized [1- 13C]pyruvate and 13C magnetic resonance spectroscopic imaging. Magn Reson Med 65: 557–563. 21264939 10.1002/mrm.22698 PMC3690628

[12] Dafni H, Larson PEZ, Hu S, Yoshihara HAI, Ward CS, et al (2010) Hyperpolarized 13C Spectroscopic Imaging Informs on Hypoxia-Inducible Factor-1 and Myc Activity Downstream of Platelet-Derived Growth Factor Receptor. Cancer Research 70: 7400–7410. 20858719 10.1158/0008-5472.CAN-10-0883 PMC2948586

[13] Warburg O (1956) On the origin of cancer cells. Science 123: 309–314. 13298683 10.1126/science.123.3191.309

[14] Vander Heiden MG, Cantley LC, Thompson CB (2009) Understanding the Warburg Effect: The Metabolic Requirements of Cell Proliferation. Science 324: 1029–1033. 19460998 10.1126/science.1160809 PMC2849637

[15] Fritz V, Fajas L (2010) Metabolism and proliferation share common regulatory pathways in cancer cells. Oncogene 29: 4369–4377. 20514019 10.1038/onc.2010.182 PMC3004916

[16] Golman K, Zandt RI, Lerche M, Pehrson R, Ardenkjaer-Larsen JH (2006) Metabolic imaging by hyperpolarized 13C magnetic resonance imaging for in vivo tumor diagnosis. Cancer Res 66: 10855–10860. 17108122 10.1158/0008-5472.CAN-06-2564

[17] Ardenkjaer-Larsen JH, Fridlund B, Gram A, Hansson G, Hansson L, et al (2003) Increase in signal-to-noise ratio of >10,000 times in liquid-state NMR. Proc Natl Acad Sci U S A 100: 10158–10163. 12930897 10.1073/pnas.1733835100 PMC193532

[18] Heimann R, Hellman S (2000) Individual characterisation of the metastatic capacity of human breast carcinoma. Eur J Cancer 36: 1631–1639. 10959049 10.1016/s0959-8049(00)00151-9

[19] Kurtz JM (2006) Local therapy, systemic benefit: challenging the paradigm of biological predeterminism. Clin Oncol (R Coll Radiol) 18: 162–165. 16605046 10.1016/j.clon.2005.11.007

[20] Tinkey PT, Milas M, Pollock RE (1999) Reliable establishment of human sarcoma xenografts in the nude rat. Sarcoma 3: 129–133. 18521275 10.1080/13577149977767 PMC2395418

[21] Howard RB, Chu H, Zeligman BE, Marcell T, Bunn PA, et al (1991) Irradiated nude rat model for orthotopic human lung cancers. Cancer Res 51: 3274–3280. 2040002

[22] Arbiser JL, Moses MA, Fernandez CA, Ghiso N, Cao Y, et al (1997) Oncogenic H-ras stimulates tumor angiogenesis by two distinct pathways. Proceedings of the National Academy of Sciences of the United States of America 94: 861–866. 9023347 10.1073/pnas.94.3.861 PMC19604

[23] Tei K, Kawakami-Kimura N, Taguchi O, Kumamoto K, Higashiyama S, et al (2002) Roles of cell adhesion molecules in tumor angiogenesis induced by cotransplantation of cancer and endothelial cells to nude rats. Cancer Research 62: 6289–6296. 12414659

[24] Woo M, Nordal R (2006) Commissioning and evaluation of a new commercial small rodent x-ray irradiator. Biomedical Imaging and Intervention Journal 2. 10.2349/biij.2.1.e10 PMC3097609 21614214

[25] Kohler SJ, Yen Y, Wolber J, Chen AP, Albers MJ, et al (2007) In vivo 13 carbon metabolic imaging at 3T with hyperpolarized 13C-1-pyruvate. Mag Res Med 58: 65–69. 10.1002/mrm.21253 17659629

[26] Hurd RE, Yen Y-F, Tropp J, Pfefferbaum A, Spielman DM, et al (2010) Cerebral dynamics and metabolism of hyperpolarized [1-13C]pyruvate using time-resolved MR spectroscopic imaging. Journal of cerebral blood flow and metabolism: official journal of the International Society of Cerebral Blood Flow and Metabolism 30: 1734–1741. 10.1038/jcbfm.2010.93 PMC2975615 20588318

[27] Wilson DM, Keshari KR, Larson PE, Chen AP, Hu S, et al (2010) Multi-compound polarization by DNP allows simultaneous assessment of multiple enzymatic activities in vivo. J Magn Reson 205: 141–147. 20478721 10.1016/j.jmr.2010.04.012 PMC2885774

[28] Cunningham CH, Chen AP, Lustig M, Hargreaves BA, Lupo J, et al (2008) Pulse sequence for dynamic volumetric imaging of hyperpolarized metabolic products. J Mag Reson 193: 139–146. 10.1016/j.jmr.2008.03.012 PMC3051833 18424203

[29] Lau AZ, Chen AP, Hurd RE, Cunningham CH (2011) Spectral-spatial excitation for rapid imaging of DNP compounds. NMR Biomed 24: 988–996. 21751271 10.1002/nbm.1743

[30] Larson PE, Kerr AB, Chen AP, Lustig MS, Zierhut ML, et al (2008) Multiband excitation pulses for hyperpolarized 13C dynamic chemical-shift imaging. J Magn Reson 194: 121–127. 18619875 10.1016/j.jmr.2008.06.010 PMC3739981

[31] Chen AP, Hurd RE, Gu YP, Wilson DM, Cunningham CH (2011) (13)C MR reporter probe system using dynamic nuclear polarization. NMR Biomed 24: 514–520. 21674653 10.1002/nbm.1618

[32] Gavrieli Y, Sherman Y, Ben-Sasson SA (1992) Identification of programmed cell death in situ via specific labeling of nuclear DNA fragmentation. The Journal of cell biology 119: 493–501. 1400587 10.1083/jcb.119.3.493 PMC2289665

[33] Itahana K, Campisi J, Dimri GP (2007) Methods to detect biomarkers of cellular senescence: the senescence-associated beta-galactosidase assay. Methods Mol Biol 371: 21–31. 17634571 10.1007/978-1-59745-361-5_3

[34] Lund EL, Thorsen C, Pedersen MW, Junker N, Kristjansen PE (2000) Relationship between vessel density and expression of vascular endothelial growth factor and basic fibroblast growth factor in small cell lung cancer in vivo and in vitro. Clinical cancer research: an official journal of the American Association for Cancer Research 6: 4287–4291. 11106245

[35] Robertson FM, Mallery SR, Bergdall-Costell VK, Cheng M, Pei P, et al (2007) Cyclooxygenase-2 directly induces MCF-7 breast tumor cells to develop into exponentially growing, highly angiogenic and regionally invasive human ductal carcinoma xenografts. Anticancer research 27: 719–727. 17465194

[36] Bourboulia D, Jensen-Taubman S, Rittler MR, Han HY, Chatterjee T, et al (2011) Endogenous Angiogenesis Inhibitor Blocks Tumor Growth via Direct and Indirect Effects on Tumor Microenvironment. AJPA 179: 2589–2600. 10.1016/j.ajpath.2011.07.035 PMC3204083 21933655

[37] Nicoletti I, Migliorati G, Pagliacci M, F G, Riccardi C (1991) A rapid and simple method for measuring thymocyte apoptosis by propidium iodide staining and flow cytometry. J Immunol Meth 139: 271–279. 10.1016/0022-1759(91)90198-o 1710634

[38] Gewirtz D, Holt S, Elmore L (2008) Accelerated senescence: An emerging role in tumor cell response to chemotherapy and radiation. Biochemical Pharmacology 76: 947–957. 18657518 10.1016/j.bcp.2008.06.024

[39] Ewald JA, Desotelle JA, Wilding G, Jarrard DF (2010) Therapy-Induced Senescence in Cancer. JNCI Journal of the National Cancer Institute 102: 1536–1546. 20858887 10.1093/jnci/djq364 PMC2957429

[40] Maddocks ODK, Vousden KH (2011) Metabolic regulation by p53. Journal of Molecular Medicine 89: 237–245. 21340684 10.1007/s00109-011-0735-5 PMC3043245

[41] Jones KR, Elmore LW, Jackson-Cook C, Demasters G, Povirk LF, et al (2005) p53-Dependent accelerated senescence induced by ionizing radiation in breast tumour cells. International Journal of Radiation Biology 81: 445–458. 16308915 10.1080/09553000500168549

[42] Sirover MA (2012) Subcellular dynamics of multifunctional protein regulation: Mechanisms of GAPDH intracellular translocation. J Cell Biochem 10.1002/jcb.24113 PMC3350569 22388977

[43] Sirover MA (2005) New nuclear functions of the glycolytic protein, glyceraldehyde-3-phosphate dehydrogenase, in mammalian cells. J Cell Biochem 95: 45–52. 15770658 10.1002/jcb.20399

[44] Dent P, Yacoub A, Fisher PB, Hagan MP, Grant S (2003) MAPK pathways in radiation responses. Oncogene 22: 5885–5896. 12947395 10.1038/sj.onc.1206701

