# Correction: Probing Early Tumor Response to Radiation Therapy Using Hyperpolarized [1-^13^C]pyruvate in MDA-MB-231 Xenografts

**DOI:** 10.1371/annotation/1f0b8488-a99a-4fe3-8e86-011ab0bf346d

**Published:** 2013-06-14

**Authors:** Albert P. Chen, William Chu, Yi-Ping Gu, Charles H. Cunningham

The name of the fourth author was spelled incorrectly. The correct spelling is: Charles H. Cunningham. The correct citation is: Chen AP, Chu W, Gu Y-P, Cunningham CH (2013) Probing Early Tumor Response to Radiation Therapy Using Hyperpolarized [1-13C]pyruvate in MDA-MB-231 Xenografts. PLoS ONE 8(2): e56551. doi:10.1371/journal.pone.0056551 

